# Baby Fever: Examining Parental Leave Policies and Pregnancy Accommodations on Emergency Medicine (EM) Residency and Graduate Medical Education (GME) Websites

**DOI:** 10.7759/cureus.103277

**Published:** 2026-02-09

**Authors:** Erin Hoag, Danielle Haussner, Danielle Melisiotis, Casey Morrone, Abagayle Bierowski

**Affiliations:** 1 Emergency Medicine, Thomas Jefferson University, Philadelphia, USA; 2 Emergency Medicine, Weill Cornell Medicine, New York, USA; 3 Emergency Medicine, Wake Forest School of Medicine, Winston-Salem, USA

**Keywords:** family leave, maternity leave, parental leave, pregnancy accommodation, website transparency

## Abstract

Introduction: Parental leave (PL) and maternity policies are important considerations that can influence prospective residents' selection of emergency medicine (EM) residency programs, yet little research has explored their transparency on program websites, often the first contact point for applicants. Accessibility is vital as policies vary widely, and related inquiries have been traditionally stigmatized. This study aimed to evaluate the availability of PL and pregnancy accommodation information provided on the websites of EM residency programs and affiliated graduate medical education (GME) sites.

Methods: Descriptive statistics were collected from 285 EM program websites and their affiliated GME websites in July 2024. Chi-square tests assessed whether PL information availability was associated with program director sex, program size, or program age.

Results: Twenty-nine program websites (10.2%) contained PL information: 16 (5.6%) detailed specific policies, and 13 (4.6%) mentioned leave. Two (0.7%) detailed specific accommodations for pregnant residents. Sixty-two program websites (21.8%) linked to a GME website containing leave information. On GME websites, 149 programs (52.3%) had PL information; 94 provided details about compensation and leave length. About 130 programs (37.5%) had no relevant information available on either site. Larger (>11 annual positions) and older (est. 2010 or earlier) programs were more likely to provide PL information (χ² (1, N = 285) = 5.91, p = 0.015; χ² (1, N = 285) = 5.95, p = 0.015)). We found no significant association between program director sex, program length, or program region and the presence of PL information on EM program or GME websites.

Conclusion: Our findings reveal significant gaps in the availability of PL and pregnancy accommodation information across EM and GME program websites, underscoring the necessity for all medical specialties to improve transparency and accessibility. Providing clear and reliable information is crucial to support prospective residents who may be hesitant to inquire about these policies during interviews. Enhancing these resources will contribute to a more inclusive and supportive training environment, ultimately benefiting both residents and program leadership.

## Introduction

Parental leave (PL) and pregnancy accommodations are increasingly recognized as essential components of trainee well-being and institutional equity in graduate medical education (GME). Before 2021, a study of the policies of 24 American Board of Medical Specialties (ABMS) organizations found that not all specialty boards outline family leave policies and that many policies had vague language [1]. In 2021, the ABMS mandated six weeks of allowable leave during training, and in 2022, the Accreditation Council for Graduate Medical Education (ACGME) enacted a requirement that programs offer six weeks of paid medical, parental, or caregiver leave [2,3]. However, while these mandates represent meaningful progress and set minimum standards, the implementation and communication of these policies remain variable across training institutions and specialties.

Emergency medicine (EM) residency applicants frequently rely on program websites as a first point of contact and a source of critical information about training culture, benefits, and scheduling flexibility. Personal and family considerations, such as PL and scheduling accommodations, play a key role in how medical students evaluate potential programs and plan their professional paths [4]. Despite growing awareness of sex equity and wellness issues, little research has evaluated how clearly programs disclose PL and pregnancy accommodations on their websites. Research in physician assistant (PA) programs has found that very few programs provide PL information on their websites, further illustrating the gap in transparency across various healthcare training programs [5]. This lack of transparency may reinforce stigma, discourage inquiries, and ultimately affect where applicants choose to train.

This study aims to assess the availability and quality of PL and accommodation information for pregnant, postpartum, or trying-to-conceive (TTC) residents on the websites of all ACGME-accredited EM residency programs and their associated GME sites as of July 2024. In addition to descriptive analysis, program-level characteristics such as size, age, geographic region, and program director sex were assessed for their association with the likelihood of publicly providing this information online.

This article was presented as a poster at the 2025 ACGME Annual Educational Conference on February 20, 2025; as an oral abstract at CORD's 2025 Academic Assembly on March 5, 2025; and as an oral abstract at SAEM's (Society for Academic Emergency Medicine) Annual Meeting on May 16, 2025.

## Materials and methods

A cross-sectional content analysis was conducted in July 2024 to evaluate the availability and quality of PL and pregnancy accommodation information on the websites of all ACGME-accredited EM residency programs and their affiliated institutional GME sites. A total of 285 EM residency programs were identified using the publicly available AAMC ERAS (Association of American Medical Colleges - Electronic Residency Application Service) database, and each program website was accessed either through the ERAS link or located via direct search combining the institution name and "emergency medicine residency program." Two independent reviewers from the research team systematically examined each EM-specific website for the presence of any PL or pregnancy accommodation information, searching all trainee-facing pages, including program overview, benefits, and policy sections, for PL or pregnancy-related content. Standardized search terms included “parental leave,” “maternity leave,” “paternity leave,” “family leave,” “pregnancy,” “lactation,” and “accommodations.” PL information was classified as specific if it included, at a minimum, both the duration of leave and whether the leave was paid or unpaid; mentions of parental or family leave without these elements were classified as vague. If no relevant information was identified after review of all available sections, the EM-specific website was coded as having no publicly available information. Following this, the associated GME website for each program was also reviewed, regardless of whether it was linked from the EM site, using the same data collection process. Any discrepancies between the two primary reviewers were resolved by a third member of the research team.

Program size and age were characteristics included in the analysis. Size was categorized as small (10 or fewer interns per year) or large (11 or more), based on the median class size calculated using data from EM-specific websites, Doximity, and EMRA Match. Age was categorized as new (established after 2007) or old (2007 or earlier), with founding year information obtained from Doximity. Additional variables collected from program-specific websites included program length (three- or four-year format), program director sex (male or female), geographic region (Northeast, Midwest, South, or West, based on Centers for Disease Control and Prevention (CDC) classifications), and primary training site type (academic or community) (Table 1). These descriptors, including program director sex, were included as exploratory program-level characteristics for descriptive purposes only and were not intended to imply individual influence or causal relationships with website content.

**Table 1:** Characteristics of ACGME-accredited emergency medicine residency programs included in analysis (N = 285) ACGME: Accreditation Council for Graduate Medical Education.

Variables	Definition	N	%
Program size	Small: 10 or fewer residents per class	144	50.5
	Large: 11 or more residents per class	141	49.5
Program age	New: established after 2006	148	51.9
	Old: established in 2006 or earlier	137	48.1
Program length	3-year format	233	81.8
	4-year format	52	18.2
Program director sex	Female	88	30.9
	Male	197	69.1
Geographic region	Northeast (CT, ME, MA, NH, NJ, NY, PA, RI, VT)	88	30.9
	Midwest (IL, IN, IA, KS, MI, MN, MO, NE, ND, OH, SD, WI)	71	24.9
	South (AL, AR, DE, DC, FL, GA, KY, LA, MD, MS, NC, OK, SC, TN, TX, VA, WV, PR)	90	31.6
	West (AK, AZ, CA, CO, HI, ID, MT, NV, NM, OR, UT, WA, WY)	36	12.6
Primary training site	Academic	226	79.3
	Community	59	20.7

Descriptive statistics were used to report the frequency and proportion of programs with relevant PL or accommodation information. Chi-square tests of independence were performed to assess associations between program characteristics and the presence of such information on either EM or GME websites. Statistical analyses were conducted using SPSS version 26 (IBM Corp., Armonk, NY), with statistical significance defined as p < 0.05. As the study relied entirely on publicly available, non-identifiable data, it was deemed exempt from institutional review board oversight.

## Results

Of the program-specific websites of the 285 ACGME-accredited EM residency programs as of July 2024, only 29 (10.2%) contained any PL information: 16 (5.6%) included specific details such as duration and pay, while 13 (4.6%) mentioned leave without elaboration. Only two EM websites (0.7%) addressed specific accommodations for pregnant, postpartum, or TTC residents (Table 2).

**Table 2:** Existing website language on pregnancy, postpartum, and fertility accommodations from EM residency programs IVF: In vitro fertilization; EM: Emergency medicine.

Institution	Policy
University of Michigan	“During the last trimester of pregnancy, and for 2 months postpartum, overnight call will not be scheduled and duty will be limited to 12 consecutive hours.” [6]
Cook County Health	“Pregnant residents are not scheduled to work night shifts or placed on rotations that take call during the first and third trimesters.” [7]
	“Given the unpredictable time requirement associated with IVF therapy, residents undergoing IVF will be given the option to activate back-up call in order to complete their full course of treatments. Rather than following the standard payback policy, activated residents are reimbursed at the internal moonlighting rate for any and all required activations.” [7]

**Figure 1:**
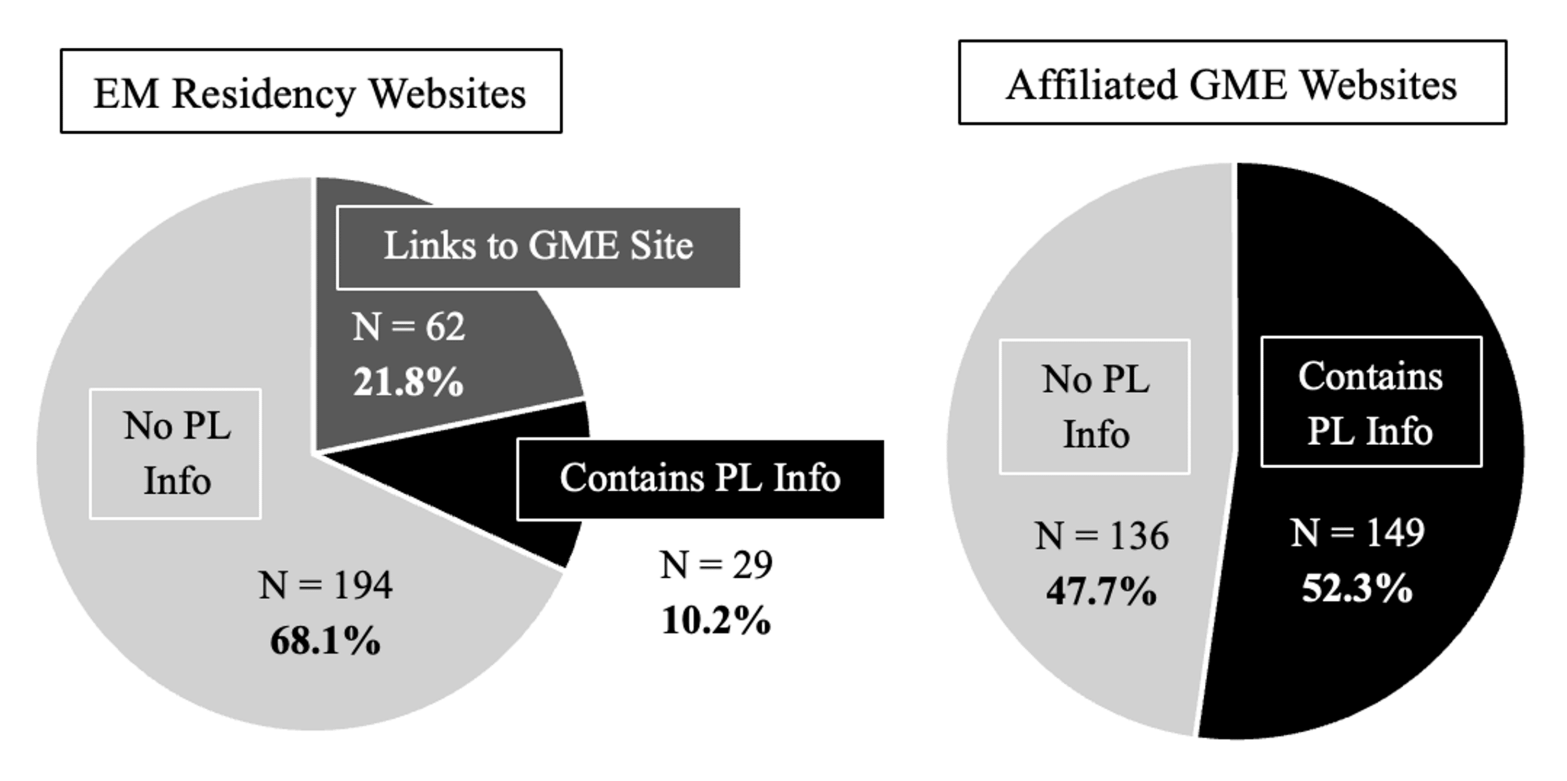
Distribution of parental leave (PL) information on emergency medicine (EM) residency program and affiliated graduate medical education (GME) websites

An additional 62 programs (21.8%) linked directly from their EM page to a GME site with relevant policy information (Figure 1).

Across all GME sites, 149 programs (52.3%) had some PL information available (Figure 1). Of these, 94 programs provided detailed information about leave length and pay, while 54 offered vague mentions without specifics. However, 130 programs (37.5%) had no PL or pregnancy accommodation information available on either their EM or GME website.

**Figure 2:**
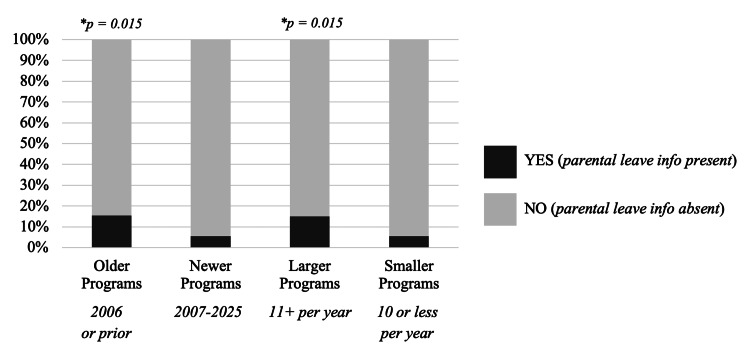
Association between program age and size and the presence of parental leave information on emergency medicine (EM) residency websites

Chi-square analyses revealed significant associations between certain program characteristics and the presence of information. Larger programs (>10 residents per class) were significantly more likely to provide PL information on their EM-specific website (χ² (1, N = 285) = 5.91, p = 0.015), with 15% of large programs doing so, compared to only 6% of small programs. Similarly, older programs (established 2006 or earlier) were significantly more likely to have both general (χ²(1, N = 285) = 6.00, p = 0.014) and any PL information (χ²(1, N = 285) = 5.95, p = 0.015) on their EM websites (Figure 2).

No significant associations were found between program director sex, program length, or geographic region and the presence of PL information. While regional variation existed in the specificity of GME site content, these findings were inconsistent and often statistically nonsignificant.

## Discussion

Providing clear and accessible leave policy information is an opportunity for programs to demonstrate institutional values around inclusion, wellness, and transparency. Despite the ACGME’s 2022 requirement that all accredited programs provide at least six weeks of paid leave for qualifying medical, parental, or caregiver needs, fewer than 11% of EM program websites included any information about parental leave, and only two contained any information about policies supporting pregnant, postpartum, or TTC residents. Even when affiliated GME websites were considered, over one-third of programs lacked relevant information entirely. This lack of transparency may place applicants in the difficult position of having to ask about stigmatized policies during the interview process, potentially influencing their sense of belonging, how they rank programs, and even how programs perceive or rank them in return.

Residency applicants frequently consult program websites when determining where to apply or interview, particularly when seeking details about scheduling flexibility and family accommodations. While data specific to EM applicants remains limited, literature from other specialties provides valuable insights into the weight trainees place on PL when selecting a program. In a national survey of pediatric residents with children, the majority reported that PL policies were a very important factor in their residency choice [8]. Similarly, Blair et al. found that approximately 40% of GME trainees planned to have children during training, and women were significantly more likely than men to consider pregnancy and childbirth in their decisions about program selection, training completion, and additional degrees [9]. These findings suggest that EM applicants may hold similar priorities, underscoring the value of making leave policies visible and accessible to reduce stigma, alleviate applicant anxiety, and support informed decision-making.

Although limited research exists on whether medical students explicitly prefer to access PL information via residency program websites, findings from Kraus et al. suggest a strong desire for transparency. In their study, 92% of applicants reported wanting PL policies to be formally addressed during the interview process, and over 60% anticipated having a child during residency [10]. Recent research in surgical residencies has also emphasized the need for standardization and transparency regarding paternity leave across specialties, which could serve as a model for EM and other fields in addressing family leave issues more comprehensively [11,12]. Given these findings, it is reasonable to infer that a similar, if not greater, proportion of applicants would value access to this information in advance through program websites. Presenting program policies both online and during interviews could serve dual purposes: reducing stigma around family planning in medicine and reinforcing a program’s commitment to wellness, transparency, and inclusivity.

Further, the emotional and mental health implications of PL policies cannot be overstated, as evidenced by the growing body of literature on postpartum depression among medical students. Research has highlighted the significant need for emotional support, transparency in leave policies, and accommodations such as breastfeeding support for students navigating pregnancy and parenthood during their training [13].

This study found that larger and older programs are more likely to include PL information on their EM-specific websites, suggesting that institutional maturity or resource availability may play a role in public policy transparency. Interestingly, the sex of the program director sex was not significantly associated with the presence of leave information, though whether a program director has children of their own may be a more relevant variable to examine. While no consistent regional trends were observed in the presence of specific or detailed information, minor geographic differences in vague policy references may reflect broader regional differences in institutional culture or state-level requirements.

While the ACGME’s 2022 policy mandating six weeks of paid PL marks a significant step forward, the visibility and accessibility of these policies on program websites remain critical. Transparent online information allows applicants to make informed decisions without needing to rely on assumptions or ask potentially stigmatizing questions. Publicly available policies also promote consistency and accountability across institutions by helping applicants understand how national requirements are enacted locally. Moreover, the culture around PL in medicine has historically been marked by stigma and silence; making information easily accessible can reduce applicant anxiety and foster a more inclusive environment. Individual programs may also offer additional benefits or accommodations beyond the ACGME minimum, such as options for modified scheduling, and website transparency helps applicants understand these nuances. Table 3 outlines suggested elements that residency programs could include on their websites to improve clarity, consistency, and applicant support.

**Table 3:** Suggested content for residency program websites on parental leave and accommodations [14,15]

Category	Recommended Website Content
Pregnancy accommodations	Clearly describe available scheduling modifications for pregnant residents, including options to avoid night shifts or 24-hour call during the first (up to 13 weeks) and third (27 weeks and onward) trimesters. Consider noting the use of a designated “baby call” or “baby jeopardy” system: an additional layer of backup coverage specifically assigned to support a pregnant resident approaching her due date, to reduce reliance on standard call pools, and to minimize last-minute disruptions. Detail procedures for requesting accommodations and timelines for doing so.
Postpartum accommodations	Specify policies on postpartum schedule flexibility, including options for shift spacing, reduced night shifts, and designated weeks for lighter schedules. Include information about lactation accommodations, such as private pumping spaces and protected time for pumping.
Accommodations for residents trying to conceive (TTC)	Acknowledge the unique needs of residents undergoing fertility treatment, including flexibility for appointments, procedures, and emotional recovery. Clarify that such absences may be treated similarly to medical appointments and that covering residents may be reimbursed accordingly (if possible).
Parental leave information	Include clear, specific details on the number of paid leave weeks provided, in accordance with ACGME policy. Clarify how the leave is structured (e.g., continuous weeks and partial return options) and if any supplemental institutional policies apply. When possible, provide examples of how schedules may be adjusted to accommodate leave - such as shifting elective or administrative rotations, front (or back) loading lighter rotations, or completing non-clinical work (e.g., research or admin blocks) remotely. Offer transparency about how leave affects scheduling, graduation timelines, and benefits eligibility.

Ultimately, when residency programs clearly communicate PL policies on their websites, they demonstrate not only regulatory compliance but also a broader commitment to supporting resident well-being and work-life balance.

Future research should explore how PL and accommodation policies are conveyed, interpreted, and valued beyond program websites. Qualitative interviews with program leaders and applicants could provide deeper insight into institutional communication practices and applicant perceptions. Additional studies should examine how the visibility of these policies influences residency choice, perceived program culture, and downstream impacts on resident well-being, inclusion, and equity, particularly for those who are pregnant, postpartum, or TTC.

Limitations

Several limitations should be considered. Website content is inherently dynamic and may have changed after the time of data collection. Additionally, programs may offer robust PL or accommodation policies that are shared through internal channels or only during interviews, which this review could not capture. However, this study intended to evaluate publicly available information (i.e., what applicants can access independently) rather than internal resources. The findings do not assess actual leave utilization, trainee satisfaction, or outcomes but rather speak to accessibility and transparency. Finally, when analyzing program director gender, we relied on the binary biological sex presented on program websites and other online sources; the authors acknowledge that this approach may not accurately reflect individual gender identity and represents a limitation of the study.

## Conclusions

Despite ACGME mandates that guarantee at least six weeks of paid PL, most EM residency and GME program websites still lack clear, detailed information on PL and pregnancy accommodations, leaving applicants to raise sensitive questions during interviews. Improving the visibility of these policies would demonstrate institutional support for trainee well-being, normalize parenthood during training, and foster greater equity in the residency selection process. As more residents pursue parenthood or fertility treatment, a transparent online policy becomes not just a regulatory requirement but also a hallmark of an inclusive and forward-thinking training environment.
